# Identification of novel genomic variants in diabetic nephropathy patients using whole-exome sequencing: a pilot investigation

**DOI:** 10.3389/fendo.2026.1798640

**Published:** 2026-03-26

**Authors:** Rashid Mir, Imadeldin Elfaki, Rehab F. Almassabi, Sanaa Almowallad, Mohamed E. Elnageeb, Hyder Osman Mirghani, Wed Albalawi, Aziz Dhaher Albalawi, Faisal H. Altemani, Jameel Barnawi, Faris J. Tayeb, Mamdoh Moawadh, Ruqaiah I. Bedaiwi, Mohammad A. Alanazi

**Affiliations:** 1 Department of Medical Lab Technology, Faculty of Applied Medical Sciences, Prince Fahd Bin Sultan Research Chair for Biomedical Research, University of Tabuk, Tabuk, Saudi Arabia; 2 Department of Biochemistry, Faculty of Science, University of Tabuk, Tabuk, Saudi Arabia; 3 Department of Medical Laboratory Sciences, College of Applied Medical Sciences, University of Bisha, Bisha, Saudi Arabia; 4 Internal Medicine and Endocrine, Department of Internal Medicine, Faculty of Medicine, University of Tabuk, Tabuk, Saudi Arabia

**Keywords:** diabetes mellitus (DM), diabetic nephropathy (DN), gene variations, genome wide association studies (GWAS), whole exome sequencing (WES)

## Abstract

**Background:**

Type 2 diabetes (T2D) is a challenge for the healthcare system. It is a metabolic disease with increased blood sugar with severe complications when it becomes uncontrolled. These complications include diabetic nephropathy (DN), neuropathy, retinopathy neuropathy, and cardiovascular diseases (CVDs). T2D is induced by genetic, environmental, and lifestyle risk factors. Therefore, it is vital to distinguish between genetic risk loci for T2D and those that specifically predispose patients to DN, which may eventually facilitate personalized risk assessment and informed genetic counseling once these markers are clinically validated. The aim of this pilot study was to examine the candidate genes associated with DN using whole-exome sequencing (WES).

**Method:**

We examined the relationship between specific genetic variations and DN in 26 (eight female and 18 male patients) clinically diagnosed DN patients in Tabuk. We utilized WES which was performed on the Illumina NovaSeq 6000 platform. Data were analyzed using an array of bioinformatic tools including FASTQC, Trimmomatic, Ensemble, Bowtie2, GATK, SAMtools, Python Comut, T2Diacod, StringDB, Gene Ontology, and KEGG.

**Results:**

The genes *THADA*, *NOTCH4*, and *TNXB* met the genome-wide threshold and showed minimal T2D association. The DN-specific genes including *SP2*, *CDH3*, and *ARFGEF2* met the suggestive DN threshold, however falling below the T2D significance. The shared genes, *INSR, HLA-DQB1*, and *CRHR1*, exhibited possible associations with T2D and DN, positioning them as key candidates for DN. Pathways and biological processes potentially affected by these gene variations include inflammatory pathway, lipid and glucose metabolism, PI3K-Akt signaling, insulin signaling, AMPK signaling pathways, and others. Moreover, we identified putative novel missense variations in the genes *SRCAP*, *PHKG2*, *TNFRSF6B*, and *PBX2*.

**Conclusion:**

Our exploratory study identified candidate novel variations in the *SRCAP*, *PHKG2*, *TNFRSF6B*, and *PBX2* genes that may be linked to DN. These results indicate that these genes are potentially involved in DN development. Nevertheless, further verifications through large-scale cohorts and functional protein studies are required to determine the clinical utility of these variants as potential biomarkers.

## 1 Introduction

Diabetes mellitus (DM) is a metabolic disease characterized by elevated blood sugar and represents an issue for the healthcare system in the Kingdom of Saudi Arabia (KSA) and worldwide (1, 2). In general, the types of DM include type 1 diabetes (T1D) which results from the auto-immune destruction of the pancreatic beta cells by T lymphocyes (3). The second is type 2 diabetes (T2D) resulting from insulin resistance and impaired function of pancreatic beta cells (4). The third type is gestational DM, developed in the late second trimester or early third trimester of gestation, in which there is also insulin resistance and defective pancreatic beta cell function (5). There is also maturity-onset diabetes of the young (MODY), which is an autosomal dominant inheritance with sound beta cell function and mutations in certain genes—for example, hepatocyte nuclear factor (HNF) and glucokinase (GCK) (6). Furthermore, there is neonatal DM caused by the mutation of the *ABCC8* or *KCNJ11* genes, leading to the abnormal development of the pancreas with altered pancreatic beta cells (7). The diagnosis of DM is achieved with a fasting plasma sugar level ≥126 mg/dL, a hemoglobin A1C (HbA1c) level ≥6.5%, a random plasma sugar ≥200 mg per dL, or a 75-g 2-h oral glucose tolerance test with a plasma glucose level ≥200 mg/dL (8). DM has socioeconomic impacts on patients and their families since it has very serious complications such as diabetic nephropathy (DN), diabetic retinopathy, diabetic neuropathy, diabetic foot, and cardiovascular diseases (CVDs) (9). DN or diabetic kidney disease (DKD) is one of the most important DM complications and represents an important cause of end-stage renal disease (ESRD) (10). Classically, the DN represented by hyperfiltration and albuminuria in the early stage; thereafter, there is a progressive deterioration of kidney function (10). The mortality rate is about 30 times in diabetic cases with nephropathy than in diabetic patients without nephropathy (10). In addition, many DN patients lose their lives because of cardiovascular complications prior to developing the ESRD (10). The screening for early DN detection is made by testing the annual albumin/creatinine ratio spot urine, and the diagnosis is verified by repeated increases of albumin secretion in the urine (11). The studies of genome-wide association and diseases association prediction have broadened our understanding of the risk loci associated with diseases such DM, CVDs, cancer, and others (12–15)—for instance, Salem et al. (16) have reported that COL4A3 missense mutation (rs55703767 G>T (Asp326Tyr) results in Alport syndrome in T1D patients (16). In addition, tripartite motif-containing 27 (*TRIM27*) and *HLA-A* genes are reported to be potential genes for DN in T1D Korean patients by Jin et al. (17). Moreover, the FERM domain containing 3 (FRMD3) rs1888747 SNP is linked with DN in different populations (18–20). Furthermore, the polymorphism of *ACE* gene producing angiotensin II was also reported to be associated with DN (21, 22). In this pilot study, we investigated the genes associated with DN in T2D patients from a Saudi population using whole-exome sequencing (WES). The identification and characterization of novel DN risk loci will assist in the prediction or detection of the susceptible individuals or populations that will help in the prevention and treatment of DN.

## 2 Materials and methods

### 2.1 Study population

This study was ethically approved by the Research Ethics Committee of the Armed Forces Hospitals, Northwestern Region (approval no. R & RE C2016-115). The cases (**Table 1**) included were with diabetic nephropathy (DN) selected from the OPD of the King Khaled Hospital in Tabuk City. They are all T2D with DN patients (26 cases, 18 male and eight female) (23) running from 40 to 60 years old. They were ethnic citizens of KSA. The inclusion criteria were DN diagnosed with elevated serum creatinine, increased estimated glomerular filtration rate (eGFR), or albuminuria. We excluded patients with other complications of DM or any significant chronic disease (e.g., malignancies or cardiovascular diseases). Blood chemistry and hematology tests were performed as part of the routine clinical procedures. All methods were performed in accordance with the relevant guidelines and regulations. All subjects provided their informed consent prior to the collection of blood samples. The socio-demographical characteristics (e.g., age, sex, and lifestyle) were recorded through a standard questionnaire.

**Table 1:** Clinical characteristics of the DN patients included in this study (*N* = 26).

Characteristics	*N* = 26^a^
Age (years)	63 (59, 66)
Age groups	
>60 years	16 (61.53%)
40–60 years	10 (38.46%)
Gender	
Female	8 (30.76%)
Male	18 (69.23%)
Duration of T2D (years)	9.00 (7.00, 10.00)
Fasting blood sugar	156 (145, 160)
HBA1c (%)	7.40 (7.05, 7.50)
HBA1c groups	
Diabetic	24 (90.30%)
Control diabetic	2 (7.69%)
Body mass index (BMI) (kg/m^2^)	27.68 (22.24, 30.05)
BMI groups	
Normal	10 (38.46%)
Obese	6 (23.07%)
Overweight	10 (38.46%)
Triglyceride (mg/dL)	210 (189, 242)
Triglyceride levels	
<150 mg/dL	2 (7.70%)
≥150 mg/dL	24 (92.30%)
Total cholesterol (mg/dL)	
<200 mg/dL	12 (46.15%)
>200 mg/dL	14 (53.84%)
HDL-C (mg/dL)	34 (29, 48)
<40 mg/dL	15 (57.69%)
≥40 mg/dL	11 (42.30%)
LDL-C (mg/dL)	
>190 mg/dl	13 (50%)
160–190 mg/dL	10 (38.46%)
100–160 mg/dL	03 (11.53%)
VLDL (mg/dL)	
>40 mg/dL	10 (38.46%)
5–40 mg/dL	16 (61.53%)
Creatinine (mg/dL)	2.50 (1.90, 2.60)
Bilirubin (mg/dL)	1 (0, 2)
AST (U/L)	30 (20, 40)
ALT (U/L)	42 (37, 48)
ALP (U/L)	97 (73, 110)
Estimated glomerular filtration rate (eGFR)	
eGFR, mL/min per 1.73 m^2^	41.0 (14.0–59.0)
Urinary albumin excretion (UAE), mg/g	90.5 (35.8–388.7)
30 mg/g	0
>30 to 300 mg/g	26
Arterial hypertension, %	90.3
Blood pressure	
Systolic blood pressure (SBP) [mmHg]	150.0 [140.3–162.0]
Diastolic blood pressure (DBP) [mmHg]	87.0 [78.0–92.0]

a Median (IQR); *N* (%).

### 2.2 Sample collection and extraction of genomic DNA

Each patient and control individual had a venipuncture to draw a 3- to 4-mL sample of peripheral blood into EDTA tubes. Using the DNeasy Blood Kit (Qiagen, Germany), genomic DNA extraction was carried out in accordance with the given manufacturer instructions. Following purification, the DNA was stored at −20°C until genotyping.

### 2.3 Whole-exome sequencing and library preparation

DNA samples from all 26 cases and one control were subjected to whole-exome sequencing (WES). Library preparation was conducted according to the protocol outlined in the Twist Human Core Exome 2.0 Kit instruction manual. Sequencing was performed on the Illumina NovaSeq 6000 platform, generating 2 × 250-base-pair (bp) paired-end reads. To ensure robust variant detection, especially for rare variants associated with T2D and diabetic nephropathy, sequencing depth was set to an average on-target depth of ~100X.

### 2.4 Quality control and preprocessing of sequencing data

Raw sequencing data quality was initially assessed using FastQC (24) to identify artifacts and overall read quality. Low-quality reads and adapter sequences were processed and removed using Trimmomatic (25). To retain only high-quality data for downstream analysis, a stringent quality cutoff was applied, and reads with a Q-score >30 were retained, while those below this threshold were filtered out. The retained high-quality reads (approximately 35 to 40 million paired-end reads per sample) were re-analyzed with FastQC to confirm the removal of all technical artifacts, thereby ensuring a robust dataset to investigate genetic variants associated with DN. These high-quality reads were mapped to the human reference genome assembly GRCh38.p14, which was downloaded from Ensembl (26). Read alignment against the reference genome was performed using Bowtie2 (27).

### 2.5 Variant calling and filtration

To ensure reliable identification and minimize false positives, two independent variant callers, GATK (28) and SAMtools (29), were employed for variant identification across all samples. This cross-validation strategy is critical to ensure robust variant calls.

The variant calling pipeline involved:

1. GATK pipeline: Utilizing HaplotypeCaller (v4.3) to simultaneously identify SNPs and small INDELs. Following GATK Best Practices for germline short variant discovery, we applied stringent hard filtering to the raw call set. The variants were filtered based on specific quality metrics: for SNPs, we required QD >2.0, FS <60.0, MQ >40.0, and SOR <3.0; for INDELs, thresholds were set at QD >2.0 and ReadPosRankSum >-20.0.
2. SAMtools Pipeline: Employing mpileup and bcftools call to independently discern SNPs and small INDELs, generating a separate variant call format (VCF) file.

Only those variants that were consistently identified by both GATK and SAMtools were retained for further analysis, which markedly enhanced the confidence in the variant set. The identified variants were primarily categorized into SNPs and insertions and deletions (INDELs). The distribution and characteristics of these retained variant classes were analyzed to identify the most prevalent types associated with T2D and DN cases. A total of 26 variant profiles were generated (26 DN cases and one healthy control). For downstream analysis, a single consolidated variant profile was created by excluding all variants shared with the healthy control sample to enrich for disease-specific genetic differences within the DN cohort.

### 2.5 Variant annotation and impact prediction

Variant annotation and functional classification were performed to prioritize biologically significant changes. The filtered VCF files were annotated using the SNPEff program, which assigns variants into functional classes based on their predicted effect, utilizing four categories of impact criteria (30). These categories include high impact (predicted to have a major functional impact, often leading to a loss-of-function, such as frameshift or nonsense variations), moderate impact (predicted to have a significant non-disruptive functional effect, such as a non-synonymous missense mutation), low impact (predicted to be mostly non-disruptive or harmless, such as a synonymous mutation), and modifier impact (predicted to have a non-coding or minimal effect, such as variants in intronic regions). This initial classification provides a general idea of possible protein-altering events. To further refine the functional effect of non-synonymous coding SNPs (which fall under the moderate impact category), SIFT (31) and the PolyPhen-2 (Polymorphism Phenotyping v2) tool were utilized (32). The integration of SnpEff impact categories with the SIFT and PolyPhen-2 score allowed for a robust strategy to prioritize variants potentially associated with T2D and DN.

To find possible new alterations in DN, we screened the variants against a wide range of public genomic databases. We classified candidates as “existing” if they were found in dbSNP, gnomAD (including Non-Finnish European, African, and Middle Eastern sub-cohorts), 1000 Genome, or ClinVar. All of the other variants, which were not in these main databases, were labeled as “novel”. To evaluate the evolutionary constraint of genes harboring candidate variants, we utilized gnomAD v4.1. Specifically, the probability of loss-of-function intolerance (pLI) score was employed to assess the depletion of predicted loss-of-function (pLoF) alleles, with a score >0.9 indicating significant intolerance to heterozygous disruption.

### 2.6 Linking WES findings to known T2D and DN associations

To contextualize the genetic variants identified through WES in our DN cohort, we mapped them against gene-level association data for T2D and DN sourced from the Type 2 Diabetes Knowledge Portal (T2DKP) (33). We visualized the association strength using a bidirectional scatter plot where the axes represent the negative log *P*-values (-log_10_*P*) for T2D and DN, respectively. Key genes were categorized and color-coded based on significance: a genome-wide threshold (-log_10_*P* ≥ 7.3) for T2D and a suggestive threshold (-log_10_*P* ≥ 2.5) for DN, allowing us to rapidly identify genes harboring patient variants with established significance in T2D only (red), DN only (blue), both (purple), or neither (gray).

### 2.7 Functional enrichment and pathway analysis

To place the identified genetic findings into a biological context, we conducted detailed functional annotation and pathway analysis specifically focusing on genes harboring high- and moderate-impact variants.

We utilized StringDB to identify protein–protein interacting genes, which aids in constructing molecular networks and inferring functional relationships among the genes harboring variants. Furthermore, the functional significance of the mutated genes was assessed using the Gene Ontology (GO) database and the Kyoto Encyclopedia of Genes and Genomes (KEGG) database, with the StringDB analysis tool used to facilitate this functional enrichment analysis within the GO and KEGG and Reactome categories. These comprehensive databases classify genes based on their established roles in critical cellular functions and metabolic pathways, enabling enrichment analysis to identify the most significantly affected biological processes and pathways, thereby linking our genetic variants to established disease mechanisms.

To control for the inflation of type I errors from multiple statistical tests, we applied the Benjamini–Hochberg false discovery rate (FDR) correction. Only pathways and biological processes with FDR-adjusted *p*-values (*p* < 0.05) were considered statistically significant, ensuring the robustness of the identified molecular signatures in the DN cohort.

### 2.8 Statistical considerations and sensitivity analysis

Because this is an exploratory study focused on rare and novel variants in a regional cohort, traditional power calculations for common-variant association (GWAS) were not applicable. A sensitivity analysis was instead conducted to define the discovery limits of the study design. With a sample of 26 patients (52 independent alleles) and a mean target coverage depth of 100X, the study achieved high technical sensitivity for variant detection. This configuration provides over 95% power to detect at least one carrier of a variant with a minor allele frequency (MAF) of ≥5.6% in this patient group. Furthermore, the 100X depth ensures high-confidence calling of ultra-rare and novel variants (MAF < 1%), minimizing false-negative results. This level of sensitivity is scientifically appropriate for a pilot investigation aiming to identify high-impact candidate genes in an understudied population.

## 3 Results

### 3.1 Clinical characteristics of the study population

The study cohort, comprising 26 patients with type 2 diabetes (T2D), presented a distinct profile marked by advanced age, male predominance, extended disease duration, and significant, multi-faceted metabolic dysregulation. The clinical characteristics, summarized in **Table 1**, paint a detailed picture of a population at a high risk for cardiovascular and renal complications.

The median age of the participants was 63 years (interquartile range [IQR]: 59, 66), with nearly two-thirds (62%) being over 60, underscoring the cohort’s older demographic. Furthermore, a substantial majority were male (69%), which is often associated with particular patterns of cardiometabolic risk. The patients had endured T2D for a median of 9.0 years (IQR: 7.0, 10.0), highlighting the chronicity of their condition. While the median body mass index (BMI) was 27.68 kg/m^2^ (IQR: 22.24, 30.05), suggesting an overweight status, the distribution across weight categories was split: 38% were overweight and 23% were obese, indicating that the majority (61%) carried excess weight.

Indicators of glycemic control revealed widespread uncontrolled DM. The median glycated hemoglobin (HbA1c) was 7.40% (IQR: 7.05, 7.50), and the median fasting blood sugar was 156 mg/dL (IQR: 145, 160). Strikingly, 92% of the participants were categorized as having frank diabetes based on their HbA1c level, demonstrating pervasive failure of long-term glycemic control. Dyslipidemia is virtually universal, creating a highly atherogenic lipid environment. The median triglyceride levels were markedly elevated at 210 mg/dL (IQR: 189, 242), with 92% of the cohort exceeding the clinical threshold of ≥150 mg/dL. Total cholesterol was ≥130 mg/dL in 100% of the patients. The median low-density lipoprotein (LDL) cholesterol was high at 190 mg/dL (IQR: 167, 216), with half (50%) of the patients exhibiting very high levels (>200 mg/dL). Conversely, protective high-density lipoprotein (HDL) cholesterol was generally low, with a median of 34 mg/dl (IQR: 29, 48), and 54% of the patients had levels <40 mg/dL. These lipid findings collectively signal a severe metabolic derangement far beyond simple hyperglycemia. Beyond glycemic and lipid anomalies, the cohort exhibited signs of target organ damage. The median creatinine levels were elevated at 2.50 mg/dL (IQR: 1.90, 2.60), hinting at a compromised renal function, a well-known complication of chronic T2D. Furthermore, elevated median liver enzymes (ALT: 42 U/L, AST: 30 U/L) suggest a degree of hepatic involvement, likely linked to the pervasive metabolic syndrome. Finally, elevated median blood pressure readings (systolic blood pressure (SBP): 132 mmHg, diastolic blood pressure (DBP): 139 mmHg) were observed, adding a hypertensive burden to the already complex clinical picture. In essence, the representative patient in this study is a 63-year-old man with nearly a decade of T2D, presenting with poorly controlled glycemia, significant abdominal obesity (overweight/obese), and a severe, multi-component dyslipidemia, alongside preliminary evidence of renal and liver dysfunction.

### 3.2 Overview of identified variants and their classification

**Figure 1:**
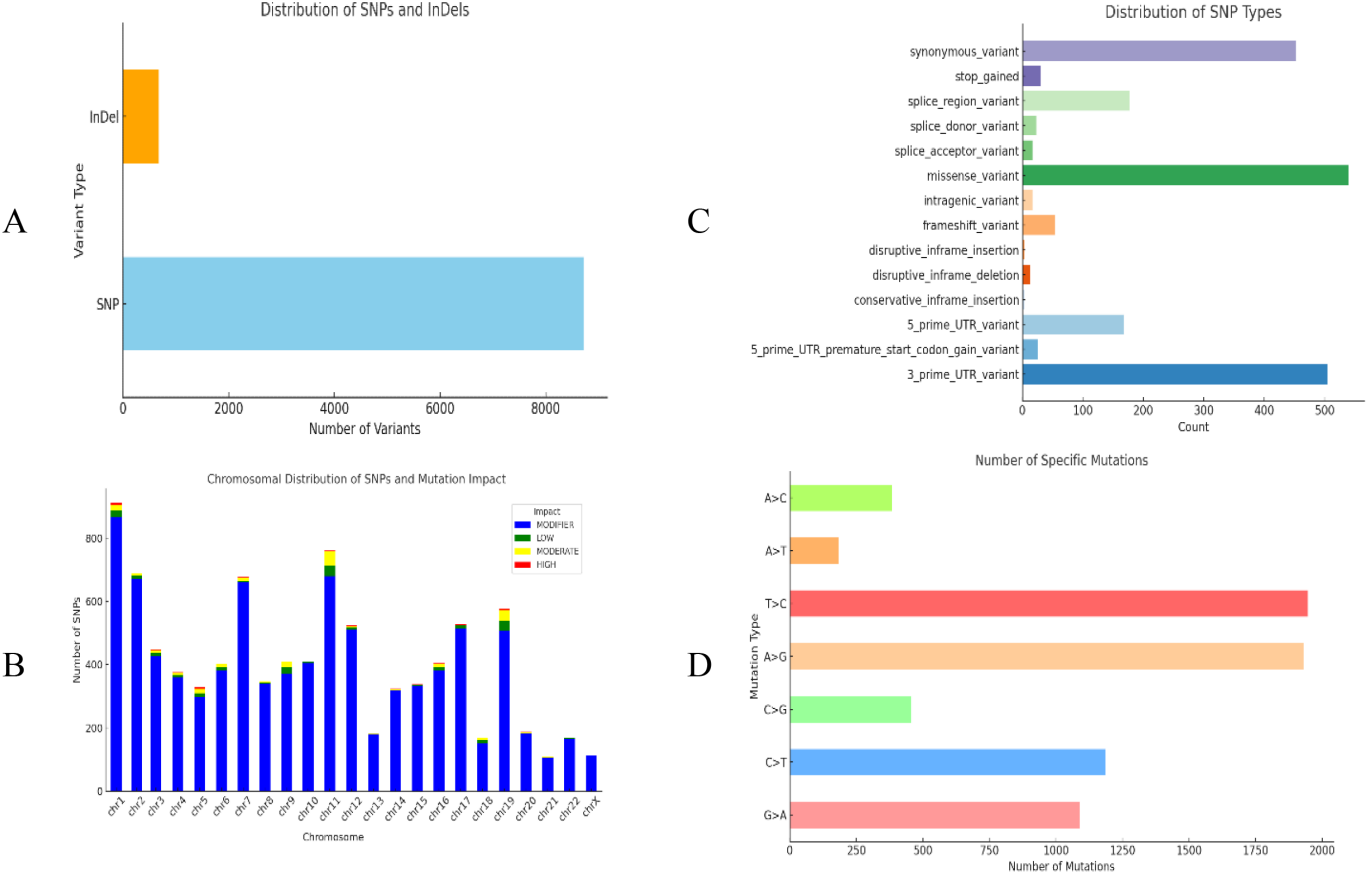
Overview of the identified variants in diabetic nephropathy compared to the control sample in a Saudi population. **(A)** Variant profile: This figure displays a summary of the total number of variants identified in the study. A total of 9,404 variants were found in diabetic nephropathy, of which 8,725 were SNPs and 679 were indels. **(B)** Chromosomal distribution of variants: This figure represents how the identified genetic variants (SNPs and indels) are distributed across different chromosomes. In this figure, chromosome 1 contained the highest number of variants. We have stacked the impact of variant on this plot. The high-impact variants (e.g., frameshift or stop-gained variations) were predominantly located on chromosome 19 (33 variants), followed by chromosome 1 (29 variants). The moderate-impact variants, such as missense variations, were most abundant on chromosome 1, with chromosomes 7 and 11 also showing substantial numbers. The low-impact variants were most frequent on chromosome 19 (95 variants), with chromosome 1 having the fewest (41 variants). Modifier variants, which are predicted to have little or no effect on protein function, were prevalent on chromosomes 1, 7, and 11. **(C)** Variant classification: This figure shows the functional classification of the variants identified using the SNPEFF program. The dominant classes included missense variants, 3’ UTR variants, and synonymous variants, all of which have potential roles in altering protein structure and function. **(D)** Base substitution plot: This plot illustrates the frequency of the different types of nucleotide substitutions (such as transitions and transversions) within the dataset. The number of occurrences for each type of base substitution is shown as horizontal bars, with the x-axis representing the count of specific base substitutions.

Whole exome sequencing (WES) identified a total of 9,404 variants across the diabetic nephropathy (DN) cohort (**Supplementary File S1**), with the vast majority being single-nucleotide polymorphisms (SNPs) (8,725) and insertions/deletions (INDELs) (679) (1A). The overall transition/transversion (Ts/Tv) ratio for the identified variants was 2.4308, confirming the high quality of the WES data and aligning with the expected range for human exome studies. Mapping the SNPs to their respective chromosomes (**Figure 1**) revealed a non-uniform distribution, demonstrating both localized “hotspots” and sparse regions that reflect the complex genetic architecture underlying T2D and its complications. The maximum number of variants (4,472 SNPs) was found on chromosome 1 (**Figure 1B**), which is notable given its historical association with multiple diabetes-related loci. Functionally, the dominant variant classes were missense variants, 3′ UTR variants, and synonymous variants (**Figure 1C**), while the base substitution plot detailed the frequency of specific base changes (**Figure 1D**).

Based on the predicted impact on gene function using the SNPEff impact criteria, the variants were further categorized (**Figure 1B**). High-impact variants (e.g., frameshift or stop-gained variations) were most frequent on chromosome 19 (33 variants), followed closely by chromosome 1 (29 variants). Moderate-impact variants (e.g., missense variations) were most abundant on chromosome 1, with chromosomes 7 and 11 also carrying substantial numbers. Conversely, low-impact variants showed the highest count on chromosome 19 (95 variants) and the lowest on chromosome 1 (41 variants). The majority of the remaining SNPs were classified as modifier variants, with the highest concentrations observed on chromosomes 1, 7, and 11.

### 3.3 Bidirectional analysis of gene association strength

**Figure 2:**
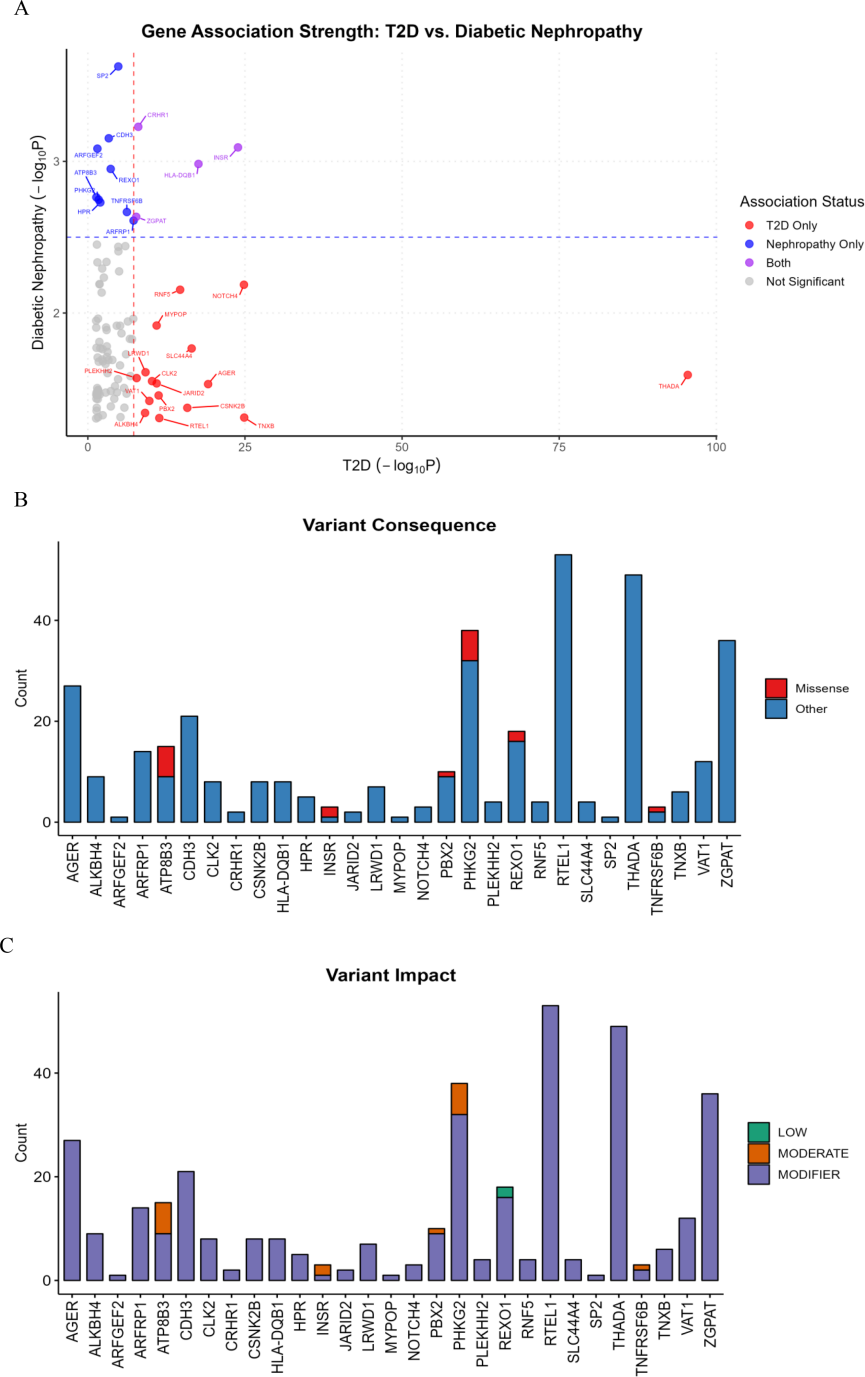
Integrative analysis of gene association strength and WES variant consequence in DN. **(A)** Gene association strength: T2D vs. DN. The plot displays the T2D (-log_10_P) association on the X-axis and DN (-log_10_P) association on the Y-axis for 98 genes mapped from the WES cohort to the T2DKP dataset. Significance thresholds delineate T2D only (red), DN only (blue), and shared **(purple)** associations. **(B)** Variant consequence. Bar plot illustrating the count of missense **(red)** versus other **(blue)** variants found in the associated genes from the WES cohort. **(C)** Variant impact. Bar plot illustrating the count of low **(green)** versus moderate **(orange)** versus modifier **(purple)** impact of variants found in the associated genes from the WES cohort.

The bidirectional analysis successfully mapped 98 genes from our WES data to the T2DKP association list (**Figure 2A**), revealing a partial genetic overlap between T2D and DN. While most genes showed low association strength, the analysis identified distinct clusters. T2D-specific genes (red), such as *THADA* (-log_10_*P* = 95.5), *NOTCH4* (-log_10_*P* = 24.8), and *TNXB* (-log_10_*P* = 24.9), met the genome-wide threshold but showed minimal DN association. DN-specific genes (blue), including *SP2* (-log_10_*P* = 3.6), *CDH3* (-log_10_*P* = 3.2), and *ARFGEF2* (-log_10_*P* = 3.1), met the suggestive DN threshold while falling below the T2D significance. The shared genes (purple), such as *INSR* (T2D: -log_10_*P* = 23.9, DN: -log_10_*P* = 3.1), *HLA-DQB1* (T2D: -log_10_*P* = 17.6, DN: -log_10_*P* = 3.0), and *CRHR1* (T2D: -log_10_*P* = 8.0, DN: -log_10_*P* = 3.2), exhibited significant or suggestive associations with both phenotypes, positioning them as key candidates for the shared genetic etiology linking primary diabetes to its renal complication.

To categorize the functional importance of variants in genes associated with T2D, DN, or both, we classified them based on consequence (**Figure 2B**) and impact (**Figure 2C**). Most variants were categorized as “others” (e.g., intronic, 5′ UTR) and assigned a modifier impact, with *RTEL1* (*n* = 53) and *THADA* (*n* = 49) harboring the highest counts, suggesting a prominent role for regulatory variants. However, critical missense variants were detected in *ATP8B3* (*n* = 6), *PHKG2* (*n* = 6), *INSR* (*n* = 2), *REXO1* (*n* = 2), *PBX2* (*n* = 1), and *TNFRSF6B* (*n* = 1). Genes with these missense changes were classified as having a moderate impact, while synonymous *REXO1* variants were categorized as low impact, underscoring that while non-coding changes are prevalent, functionally relevant protein-altering variants are present in key disease-associated genes, including the pleiotropic *INSR*.

### 3.4 Prioritized novel coding variants in DN

WES reported five novel mutations among our T2D cases with nephropathy (**Table 2**). The prioritization of novel missense variants identified in our diabetic neuropathy cohort, based on *in silico* prediction of deleterious effect, highlighted five key genes (**Table 3**). All variants were classified as having a moderate impact on the resulting protein. Specifically, the variants *SRCAP* (p.D2345A) and *PBX2* (p.D365A) were predicted to be deleterious by SIFT and either possibly damaging or benign by PolyPhen. Notably, the variants in *SRCAP* and PBX2 occur in genes with a pLI score of 1.00. This maximum gene-level constraint suggests that these loci belong to a class of genes in which the human genome rarely tolerates loss-of-function (LoF) alleles, often indicating that disruptive variation—including missense changes—may have significant phenotypic consequences. The variant in *TNFRSF6B* (p.T262P) was also called deleterious by SIFT, while *PHKG2* (p.V57A) received a possibly damaging score from PolyPhen despite being tolerated by SIFT. Both genes had pLI scores of 0.00, indicating that they are generally tolerant of variation across the population. While these variants are novel in the context of the databases searched, they currently remain variants of unknown significance (VUS) according to the preliminary evidence. Based on ACMG criteria, all of these prioritized novel variants were under PM2 category (extremely low frequency in gnomAD population databases).

**Table 2:** Biological processes and pathways enriched by high- and moderate-impact variants.

Pathway or process	Term ID	Enriched genes	Category	FDR
Response to stress	GO:0006950	*PLIN3*, *CYBA*, *ALOX15*, *FASN*, *SHMT2*, *SIRT6*, *TFRC*, *IL5RA*, *LDLR*, *MAP4K1*	GO Process	4.29 × 10^-2^
Inflammatory response	GO:0006954	*CYBA*, *ALOX15*, *FASN*, *TFRC*, *IL5RA*	GO Process	4.73 × 10^-2^
Fibroblast growth factor receptor signaling pathway	GO:0008543	*FGFR3*	GO Process	6.95 × 10^-6^
Positive regulation of metabolic process	GO:0009893	*CYBA*, *INSR*, *EEF2*, *SIRT6*, *FGFR3*, *ALPL*, *TFRC*, *LDLR*, *PHKG2*	GO Process	1.84 × 10^-2^
Regulation of lipid metabolic process	GO:0019216	*SIRT6*, *FGFR3*, *LDLR*	GO Process	4.80 × 10^-2^
Phosphate-containing compound metabolic process	GO:0006796	*ALOX15*, *INSR*, *FGFR3*, *ALPL*, *PHKG2*, *MAP4K1*	GO Process	1.15 × 10^-2^
Bone mineralization	GO:0030282	*ALOX15*, *FGFR3*, *ALPL*	GO Process	1.40 × 10^-3^
Glucose homeostasis	GO:0042593	*CYBA*, *INSR*, *SIRT6*	GO Process	6.40 × 10^-3^
Regulation of MAPK cascade	GO:0043408	*ALOX15*, *INSR*, *FGFR3*, *MAP4K1*	GO Process	1.95 × 10^-5^
MAPK signaling pathway	hsa04010	*INSR*, *FGFR3*, *MAP4K1*	KEGG	9.28 × 10^-8^
Ras signaling pathway	hsa04014	*INSR*, *FGFR3*	KEGG	3.22 × 10^-14^
HIF-1 signaling pathway	hsa04066	*INSR*, *TFRC*	KEGG	2.04 × 10^-5^
PI3K-Akt signaling pathway	hsa04151	*INSR*, *FGFR3*	KEGG	3.07 × 10^-7^
AMPK signaling pathway	hsa04152	*INSR*, *FASN*, *EEF2*	KEGG	3.83 × 10^-5^
JAK-STAT signaling pathway	hsa04630	*IL5RA*	KEGG	2.02 × 10^-2^
Insulin signaling pathway	hsa04910	*INSR*, *FASN*, *PHKG2*	KEGG	2.53 × 10^-10^
Membrane trafficking	HSA-199991	*PLIN3*, *TFRC*, *LDLR*	Reactome	1.88 × 10^-2^
Clathrin-mediated endocytosis	HSA-8856825	*TFRC*, *LDLR*	Reactome	9.37 × 10^-5^

This table summarizes the top GO, KEGG, and Reactome terms identified in the functional enrichment analysis, highlighting genetic links to core pathways involved in metabolic dysregulation, stress response, and inflammation.

**Table 3:** Prioritized novel missense variants identified in DN cases.

Gene	HGVSc	HGVSp	Consequence	Impact	SIFT	PolyPhen	pLI	ACMG classification
*SRCAP*	NM_006662.3:c.7034A>C	NP_006653.2:p.D2345A	Missense variant	Moderate	Deleterious	Possibly damaging	1.00	VUS
*PHKG2*	NM_000294.3:c.170T>C	NP_000285.1: p.V57A	Missense variant	Moderate	Tolerated	Possibly damaging	0.00	VUS
*TNFRSF6B*	NM_003823.4:c.784A>C	NP_003814.1:p.T262P	Missense variant	Moderate	Deleterious	Benign	0.00	VUS
*PBX2*	NM_002586.5:c.1094A>C	NP_002577.2:p.D365A	Missense variant	Moderate	Deleterious	Benign	1.00	VUS

### 3.5 Functional enrichment analysis

The functional enrichment analysis of genes carrying high- and moderate-impact variants (**Table 3**) revealed a strong genetic connection to the key pathological mechanisms of diabetic nephropathy (DN). The most enriched terms clustered around metabolic dysregulation and stress response, explicitly pointing to pathways like insulin signaling (KEGG: hsa04910) and glucose homeostasis (GO: 0042593), which confirms the genetic disturbance of basic glucose control. Furthermore, enrichment in the AMPK signaling pathway (KEGG: hsa04152), regulation of lipid metabolic process (GO: 0019216), and terms for inflammatory response (GO: 0006954) underscore the role of genetic variants in driving chronic inflammation, lipotoxicity, and energy-sensing failure. The analysis also highlighted pathways critical for cellular stress and structural integrity, including the HIF-1 signaling pathway (KEGG: hsa04066), phosphate-containing compound metabolic process (GO: 0006796), and bone mineralization (GO: 0030282), suggesting that genetic variants may compromise the cell’s response to hypoxia and its capacity to manage essential mineral and phosphate dynamics. There is frequent presence of genes like INSR and FGFR3 across major signal transduction cascades, including the MAPK and PI3K-Akt signaling pathways.

## 4 Discussion

Type 2 diabetes (T2D) is induced by the interaction of the genetic and environmental risk factors (34). DN is one of the serious complications of DM. In this pilot study, we examined the association of gene variations with DN in T2D patients from the Tabuk population.

### 4.1 The gene variations in diabetic nephropathy patients

The *CRHR1* (corticotropin-releasing hormone receptor 1) gene showed variations (**Figure 2**). Our results indicated that this gene showed suggestive associations with T2D and DN. CRHR1 enhances the glucose-stimulated secretion of insulin (35, 36). *CRHR1* gene variants can result in hypercortisolism, resulting in dysfunction of serotonin and depression, which can contribute to increased blood sugar, insulin insensitivity, and visceral obesity; these are T2D characteristics (36).

*HLA-DQB1* (major histocompatibility complex, class II, DQ beta 1) also showed gene variations with possible association with DM and DN (**Figure 2**). It is an important part of the immune system. Polymorphisms of the *HLA-DQB1* genes may be associated with autoimmune diseases such as T1D, multiple sclerosis, celiac disease (37), and renal function (38). This result is consistent with a study by Jin et al., 2023 (17) conducted in a Korean population, which reported that *HLA-DQB1* gene variations are involved in the etiology of DN.

The *ZGPAT* (zinc finger CCCH-type and G-patch domain containing) gene also showed variations (**Figure 2**) with potential linkage with T2D and DN (**Figure 2**). It is a transcription factor that potentially regulates the transcription of many genes (39). The results showed that there are variations in the *INSR* gene with a possible association with T2D and DN (**Figure 2**). The INSR gene encodes the insulin receptor, a transmembrane protein, important for insulin signaling pathways that are an essential part of glucose homeostasis (40). Variations in the INSR gene can lead to the development of insulin resistance and hyperglycemia (41). This gene was reported to be associated with neonatal hyperinsulinemic hypoglycemia and familial DM (42). In addition, the *INSR* (and MTOR) polymorphisms were linked with T2D and DN in the northeast Chinese Han population (43).

In addition, we also encountered variations in the *CDH3* gene (**Figure 2**). It encodes cadherin 3 (also called P-cadherin). Cadherins are transmembrane proteins in epithelial tissues and function in cell–cell adhesion, junction formation, and maintenance of tissue integrity; they are expressed in tissues such as esophagus, renal tubules, and intestines (44). We propose that the *CDH3* gene variations can contribute to DN in hyperglycemic conditions of DM. There were also variations in the *ARFGEF2* gene (encoding ARFGEF2 protein, also known as BIG2 protein). This protein is a guanine nucleotide exchange factor (GEF) that activates ADP-ribosylation factors (ARFs) (45) and is important for the integrity of the endosomal compartment (46).

Our result also indicated that there were variations in the *ARFRP1* gene (**Figure 2**). This gene encodes ARF related protein 1 (ARFRP1). This protein and another protein (GOPC) are needed for the secretion of insulin stimulated by glucose in pancreatic beta cells (47). The *SP2* gene (Sp2 transcription factor) also showed variations (**Figure 2**). *Sp2* regulates different functions of the cells—for example, cell division, proliferation, differentiation, and death (48). It has been suggested that SP2 has a role in renal growth and differentiation, and it is implicated in kidney function and diseases (49). It has been reported that the overexpression of SP2 results in the activation of the insulin signaling pathway, improves insulin resistance, and decreases oxidative stress in neurons. In addition, SP2 enhances the suppression of inflammatory response (48). Therefore, SP2 is proposed as a biological target for diabetic encephalopathy (48). The role of SP2 in insulin resistance and T2D remains to be investigated in future studies.

The results also showed that there were variations in *ATP8B3* (**Figure 2**). This gene encodes the aminophospholipid transporter (50). It is a member of P-type cation transport ATPases family, particularly to the aminophospholipid-transporting ATPases subfamily (50). The role of this gene in DN development requires further investigations. The *PHKG2* (phosphorylase kinase catalytic subunit gamma 2) gene also showed variations. This enzyme has a role in the activation of glycogenolysis. Variants in the *PHKG2* gene are linked with liver glycogen storage disease (GSD) IX (51). The result indicated that the *THADA* gene showed variations (**Figure 2**). This gene encodes the thyroid-adenoma-associated protein. This result is in agreement with previous studies indicating the association of *THADA* with T2D development (52, 53). *THADA* is implicated in T2D via the regulation of pancreatic beta cell function and apoptosis, and it has been suggested that targeting *THADA* expression or activity is a strategy for T2D prevention and therapy (54).

*NOTCH4* (neurogenic locus notch homolog 4) also exhibits gene variations (**Figure 2**). This gene encodes NOTCH4 protein. It has been proposed that this protein modulates the PI3K/AKT-dependent insulin signaling and implicated in T2D development (55). Our results are consistent with a study by Liu et al. (56), who reported the association of *NOTCH4* gene with susceptibility to DN in a Chinese population.

In addition, the results also showed variations in *RNF5* (encodes the ring finger protein 5) (**Figure 2**). *RNF5* is a conserved E3 ubiquitin ligase involved in the elimination of the misfolded proteins from the endoplasmic reticulum (ER) via the ER-associated degradation pathway (57). In addition, RNF5 negatively regulates ER stress (57). ER stress is an important contributor to T2D development (58), the role of *RNF5* genes in DN requires future investigations. The *TNXB* gene also showed variations (**Figure 2**). It encodes tenascin-X (TNX), a large ECM glycoprotein widely expressed in various organs such as the heart, lungs, and kidney (59, 60). The dysfunction of TNX is an associated disorder of connective tissues (60). TNXB gene variants are reported to cause single kidney agenesis (59); however, its role in DN needs further studies. The *SLC44A4* (solute carrier family 44 member 4) gene also varied. This gene encodes choline transporter-like protein 4. The *SLC44A4* gene was significantly correlated with the stage of clear cell renal cell carcinoma (61).

Our results indicated that there were variations in the *AGER* gene (**Figure 2**). This gene encodes advanced glycosylation end product (AGE) receptor. It is a transmembrane protein and belongs to the immunoglobulin superfamily expressed in pancreatic beta cells, WBCs, nerve cells, lung, liver, smooth muscles, and brain (62). It functions as a scavenger and mediates intracellular signaling (63). The SNPs of the *AGER* gene are linked to DM and DM complications (63, 64). In hyperglycemic conditions, it acts at the levels of gene transcription to release different proinflammatory agents (63), which may contribute to DN development. This result is in line with a previous study conducted on a Chinese population (56). The *CLK2* gene also exhibits variations (**Figure 2**). This gene encodes a protein kinase catalyzes the phosphorylation of ser/thr- and tyr-containing substrates and called Cdc2-like kinase 2 (CLK2). CLK2 is involved in the regulation of the metabolism of fatty acids (65). CLK2 also suppresses the expression of gluconeogenic genes and is activated by insulin/Akt signaling (65).

The results showed that the *JARID2* gene also showed variations. This gene is required for the late-stage differentiation of pancreatic beta cells in embryonic life (66), and ARID2 promoted glycolysis and lipid metabolism in tumor cells. Nevertheless, its precise role in DM and DN needs further studies. In addition, the results indicated that the *RTEL1* gene (encodes a helicase that regulates the elongation of telomeres) also exhibits gene variations (**Figure 2**). The *RTEL1* gene variations can be associated with renal fibrosis (67). The *REXO1* (RNA exonuclease 1 homolog) gene also varied in our samples (**Figure 2**). It is a cofactor involved in gene regulation and can be implicated in diabetes (68). Nevertheless, the exact role has yet to be examined.

The results also showed that there were gene variations in the *ALKBH4* gene (**Figure 2**). This gene encodes alkB homolog 4, lysine demethylase. This protein has different functions—for instance, it regulates the dynamics of actomyosin in processes such as cytokinesis and cell migration; it is also involved in transcription (69). In addition, there were novel missense variations in the *SRCAP* gene (**Table 3**). SRCAP encodes the SNF2-related CREBBP activator protein (70). *SRCAP* gene mutations result in delayed bone maturation, expressive-language problem, distinctive features of the face, and polycystic kidneys (70, 71). The possible role of the *SRCAP* gene in DN has yet to be investigated.

The results also indicated that the *TNFRSF6B* (tumor necrosis factor receptor (TNFR) superfamily member 6b) gene has missense variants (**Figure 2**, **Table 2**). This gene is also called DcR3 (decoy receptor 3) (72). This gene was reported to be associated with BMI, T2D (73), T1D (an autoimmune disease) (74), and CKD (75). The *PBX2* gene showed a novel missense variant (**Figure 2**, **Table 3**). The PBX proteins are conserved transcription factors and members of the superfamily of three amino acid loop extension (TALE) homeodomain proteins (76). They have important roles in organ development in the early embryonic life (76), and their potential role in DM and DN has yet to be understood.

### 4.2 Gene variations in the metabolic pathways of DN patients

Our results indicated that there were variations in the insulin signaling pathway (**Table 2**). The gene variations in the insulin signaling pathway are linked to T2D (77). It has been reported that hyperglycemia impairs the insulin signaling pathway in the glomerulus, promoting a proapoptotic environment and leading to DN (78). We also found variations in genes involved in glucose metabolism in DN cases (**Table 3**). These results are in agreement with studies which reported that gene variations in the glucose metabolism pathway lead to hyperglycemia (79). Hyperglycemia alters the metabolism of podocytes by inducing DRP1-mediated mitochondrial fission via ROCK1, resulting in deleterious effects in podocytes (80). The results showed that there were variations in the oxidative stress pathway in DN cases (**Table 2**). This result is consistent with studies that reported the role of oxidative stress in DN (81, 82). We also encountered gene variations in lipid metabolism and inflammatory pathways (**Table 2**). It has been reported that lipotoxicity and low-grade systemic chronic inflammation are linked to DN (83).

The results indicated that the HIF‐1α signaling pathway is affected by the variations (**Table 2**). These results may be in agreement with a study which reported that HIF‐1α prevents tubular injury in DN through heme oxygenase‐1-mediated control of mitochondrial dynamics (84). In addition, our results also indicated gene variations in the AMPK and MAPK pathways (**Table 1**). The AMPK pathway was described as a target to control diabetes and its complications (85), while the activation of the MAPK pathway was reported to be implicated in DN and that targeting the MAPK pathway could be an effective therapy for DN (86). Moreover, membrane trafficking is affected by gene variations in DN (**Table 2**). This result is quite consistent with a study which reported that membrane trafficking is implicated in the occurrence of epithelial–mesenchymal transformation (EMT) that is associated with glomerular fibrosis—for example, the transforming growth factor beta induced EMT in DN (87).

### 4.3 Distinguishing DN-associated candidate variants from general T2D risk

A major challenge in diabetic genomics is distinguishing variants that drive the primary metabolic disease (T2D) from those that predispose patients to microvascular complications such as diabetic nephropathy (DN). As an exploratory pilot investigation, our study focused on identifying genomic variations within a clinically confirmed DN cohort. While some variants may be shared with the broader T2D background, several factors suggest that our findings are highly relevant to the renal phenotype.

The prioritized candidate genes—including SP2, CDH3, and ARFGEF2—reached suggestive significance in our DN analysis but are not typically identified as primary risk factors in large-scale T2D GWAS. This suggests a potentially complication-specific role. Furthermore, functional enrichment mapped these variants to the PI3K-Akt and AMPK signaling pathways. Unlike general T2D variants that affect insulin secretion, these pathways are central to podocyte health and the prevention of renal fibrosis.

By filtering against global databases and utilizing a control individual, we minimized population-specific background noise. While a definitive separation between T2D and DN risk requires larger longitudinal cohorts including T2D patients without renal complications, these identified candidate markers provide a biologically plausible foundation to understand the unique genomic landscape of DN in the Saudi population.

The limitations of this study include the limited sample size, its being a single-center study, and the age of onset of T2D and DN that were not included in the analyses. The early-onset T2D is linked with increased risks of DN, declined kidney function, and ESRD (88). Another limitation is that our study did not include T2D only as a control group; as this is an exploratory pilot investigation focusing on a cohort already diagnosed with DN, a primary challenge remains the definitive separation of genetic variants predisposing to T2D generally from those specifically driving renal complications. While our functional mapping points toward kidney-specific pathways, the current study design does not statistically decouple the metabolic background of T2D from DN-specific risk. Further studies with a larger sample size and in different ethnic populations are needed. In addition, protein–protein interaction and protein functional studies are required to examine the effects of these gene variations to confirm these findings (89–91). Therafter, these gene variants can be considered for genetic counselling for the prediction and early detection of DN that will help in prevention and treatment strategies.

## 5 Conclusion

To sum up, in this pilot investigation, we conducted whole-exome sequencing (WES) in patients with diabetic nephropathy (DN). The results indicated that the genes *THADA*, *NOTCH4*, and *TNXB* met the genome-wide threshold and showed a minimal type 2 diabetes (T2D) association. The DN-linked genes *SP2*, *CDH3*, and *ARFGEF2* met the suggestive DN threshold, however falling below the T2D significance. The genes *INSR*, *HLA-DQB1*, and *CRHR1* exhibited possible associations with T2D and DN, making them important candidates for DN development. We also detected novel missense variations in the genes *SRCAP*, *PHKG2*, *TNFRSF6B*, and *PBX2*. The pathways and biological processes possibly influenced by these gene variations include inflammatory pathway, lipid and glucose metabolism, PI3K-Akt signaling, insulin signaling, and AMPK signaling pathways. These results require further verification in future case–control, genomic, and protein functional studies. Thereafter, these loci can be used for the prediction, prevention, and treatment of DN.

## Data Availability

The data presented in the study are deposited in the https://www.ncbi.nlm.nih.gov repository, accession number PRJNA1357043.
